# Female Reproductive System Assembloids in Health and Disease

**DOI:** 10.3390/cells15191792

**Published:** 2026-09-30

**Authors:** Mete Hakan Karalök, Burcu Biltekin, Turgay Barut, Süleyman Ergün

**Affiliations:** 1 Department of Obstetrics and Gynecology, Faculty of Medicine, Istanbul Atlas University, Istanbul 34408, Türkiye; 2 Department of Histology-Embryology, Faculty of Medicine, Istanbul Atlas University, Istanbul 34408, Türkiye; burcu.biltekin@atlas.edu.tr; 3 Yale Medicine Greenwich Hospital Fertility Center, 5 Perryridge Rd., Greenwich, CT 06830, USA; turgay.barut@greenwichhospital.org; 4 Institute of Anatomy and Cell Biology, University of Würzburg, 97070 Würzburg, Germany; 5 Atlas University Research Center (ARC), Istanbul Atlas University, Istanbul 34403, Türkiye; suleyman.ergun@atlas.edu.tr; 6 Institute of Neuroanatomy, University Hospital Hamburg-Eppendorf, 20251 Hamburg, Germany

**Keywords:** organoids, assembloids, endometrium, decidualization, implantation, ovary, fallopian tube, blastoid

## Abstract

In just over a decade, in vitro modeling of the female reproductive tract has advanced from planar monolayers of immortalized cells to self-organizing epithelial organoids and, most recently, to increasingly complex multicellular and multicompartment 3D models, including assembloids that combine organoids with additional organoid modules or specialized cell lineages and demonstrate post-assembly integration and self-organization. This review concentrates deliberately on organoid, assembloid, and related multicellular 3D models rather than on reproductive physiology per se. We first establish a criterion-based framework that distinguishes organoids, assembloids, co-cultures, organ-on-chip systems, and blastoids according to assembly strategy, cellular composition, and evidence of structural and functional integration, and trace the rapid consolidation of the assembloid concept from neuroscience into reproductive biology. We then survey, organ-by-organ and with a critical eye, what these models have demonstrated in the endometrium, the ovary and follicle, the fallopian tube, and the embryo–maternal interface, together with the enabling technologies (including microfluidics, vascularization strategies, biomaterials and bioprinting, and single-cell and spatial benchmarking) and the limitations that still constrain the field. We close with emerging translational opportunities in disease modeling, drug and toxicology screening, and personalized reproductive medicine, while highlighting the requirements for physiological validation, reproducibility, standardization, and appropriate ethical and regulatory oversight.

## 1. Introduction

The female reproductive tract is an integrated set of hormone-responsive organs whose epithelia share a common developmental origin but diverge sharply in architecture. The endometrium comprises a luminal and a glandular epithelium supported by a fibroblastic stroma that contains vascular and immune cells, including uterine natural killer (uNK) cells and macrophages [1,2,3]. The ovarian cortex houses follicles in which an oocyte is surrounded by granulosa cells and an outer theca layer. The fallopian tube presents a ciliated–secretory epithelium over a stromal and smooth-muscle wall, and the cervix a transition from glandular to squamous epithelium. Across these tissues, epithelial polarity and interactions with underlying stromal and other cellular compartments are important features for physiologically representative modeling [4,5,6,7,8,9,10,11,12,13,14].

Function across these compartments is governed by the hypothalamic–pituitary–ovarian axis and, in particular, by the cyclic interplay of estradiol (E2) and progesterone (P4). During the proliferative phase, follicle-derived E2 drives endometrial epithelial and stromal proliferation and upregulates the progesterone receptor [11,12]. Within the follicle, the two-cell/two-gonadotropin arrangement couples luteinizing-hormone-driven thecal androgen synthesis to follicle-stimulating-hormone-driven granulosa aromatisation of those androgens to estrogen, while paracrine signals such as GDF9 and BMP15 from the oocyte and AMH and kit ligand from granulosa cells coordinate folliculogenesis [14]. After ovulation, the corpus luteum secretes P4, which switches the glandular epithelium to a secretory, embryotrophic phenotype and, together with locally elevated cyclic AMP, triggers decidualization: the transformation of stromal fibroblasts into secretory decidual cells expressing prolactin and IGFBP1 [11]. Decidualization is not a purely stromal event but involves reciprocal epithelial–stromal interactions, and single-cell analyses have resolved differentiated, senescent, and progesterone-resistant stromal states associated with implantation competence and reproductive outcome [5,13]. Implantation itself unfolds within a narrow window of receptivity through apposition, adhesion, and trophoblast invasion, with decidualizing stromal cells contributing to embryo–endometrial signaling and selection [4,13]. Reproducing these multicellular and spatially organized processes remains difficult in conventional single-cell-type or two-dimensional systems.

Despite this detailed physiological picture, the mechanistic roots of major reproductive disorders remain incompletely understood. It is still unclear why implantation fails in some patients with RIF despite transfer of euploid embryos, and how embryonic and endometrial factors interact to determine implantation outcome [4]. The molecular basis of endometriosis and adenomyosis, endometrial progesterone resistance, and recurrent pregnancy loss (RPL) is only partly understood, and a substantial proportion of RPL and infertility remains unexplained [5,15]. How oocyte quality can be preserved or restored in women with diminished ovarian reserve, and how the earliest, experimentally and ethically difficult-to-access stages of human implantation and placentation are regulated, also remain major questions. Progress has been constrained in part by the limited availability of human experimental systems that reproduce the relevant cellular interactions and tissue interfaces.

Until roughly a decade ago, reproductive modeling depended heavily on several complementary but individually limited experimental approaches. Two-dimensional monolayers of primary cells or of immortalized and carcinoma-derived lines (Ishikawa, ECC-1, RL95-2, THESC) were accessible and scalable but could show altered polarity, junctional organization, and hormone-receptor expression and provided limited representation of epithelial–stromal interactions [6,7]. Molecular and biochemical assays enabled detailed interrogation of individual pathways but necessarily removed them from aspects of their multicellular tissue context. Animal models, chiefly rodents, provided in vivo physiology but differ from humans in important features of menstruation, decidualization, and the depth and invasiveness of implantation, complicating direct translation [7]. Collectively, these limitations motivated the development of more physiologically representative human, tissue-level, and hormone-responsive experimental systems.

This need has contributed to the development of New Approach Methodologies (NAMs) encompassing human-relevant in vitro and in silico approaches that can complement and, in appropriate contexts, reduce reliance on animal experimentation. Within this movement, adult-stem-cell-derived organoids restore aspects of three-dimensional epithelial architecture, polarity, differentiation, and hormone responsiveness but generally lack the stromal, immune, vascular, and other tissue compartments present in vivo. More complex reproductive 3D models address these limitations through different assembly strategies. In this review, we reserve the term “assembloid” for systems in which an organoid is combined with another organoid or with specialized cell populations and the assembled components subsequently demonstrate three-dimensional integration and self-organization. Importantly, assembloid identity does not require representation of every native lineage: epithelial–stromal systems predominate in reproductive applications, whereas immune and endothelial incorporation remains protocol-specific. Systems in which components are co-maintained without demonstrated post-assembly integration are considered separately as co-cultures or multicellular 3D models, while organ-on-chip denotes an engineering format that may incorporate any of these biological configurations. Accordingly, the sections that follow focus on organoids, assembloids, and related reproductive 3D models rather than providing a comprehensive account of reproductive physiology: Section 2 establishes their terminology and design principles, Section 3 examines their organ-specific applications, enabling technologies, and limitations, and Section 4 considers their translational outlook.

## 2. Organoids and Assembloids: Definitions, Distinctions, and Development

### 2.1. The Organoid Paradigm and Its Intrinsic Limits

The modern organoid era began with the demonstration by Sato and colleagues that single LGR5+ intestinal stem cells self-organise within a laminin-rich matrix into self-renewing epithelial structures with crypt-like and villus-like domains [16]. The principle generalized across organ systems and reached the female reproductive tract in 2017, when long-term, genetically stable, hormone-responsive endometrial epithelial organoids were established from human and mouse tissue [1,17]. Self-renewing human endometrial organoids that reproduce key features of glandular epithelium were confirmed independently, and comparable systems followed for the fallopian tube [2], ovary, cervix, and trophoblast [18,19,20,21,22,23,24,25,26,27,28,29]. Reproductive organoids recover several tissue features poorly represented in conventional monolayer culture, including glandular morphology, apico-basal polarity, cyclic responsiveness to E2 and P4, secretory output, and, in disease-derived material, patient-specific phenotypes and experimentally informative drug responses [3].

Their defining strength is also a central limitation in many reproductive applications. Reproductive epithelial organoids are self-organizing epithelial structures and therefore generally do not incorporate the stromal, immune, vascular, and other cellular compartments that influence epithelial behavior in vivo. Endometrial gland organoids therefore reproduce features of glandular epithelium but not the stromal decidualization response, uNK cell interactions, or the Wnt-, FGF-, and contact-mediated epithelial–stromal crosstalk that contributes to endometrial differentiation and receptivity [1,3,7]. Grown in basement-membrane extracts, they also commonly form cystic structures with an apical-in polarity, limiting direct access of an experimental embryo or blastoid to the apical epithelial surface [6,10]. These limitations are particularly relevant to reproductive processes that depend on interactions between multiple cellular compartments. Decidualization, receptivity, and embryo–endometrial interactions, for example, cannot be fully reconstructed in epithelial monoculture alone. Comparable compartmental gaps apply across the tract, from the granulosa–theca–oocyte unit of the follicle to the epithelial, stromal, and smooth-muscle compartments of the fallopian tube. Recognition of these limitations has driven the development of multicellular reproductive 3D models, including assembloids designed to restore specific tissue–tissue interactions.

### 2.2. The Assembloid Concept and Its Arrival in Reproductive Biology

The assembloid concept emerged from efforts to overcome the lineage and regional constraints of individual organoids by combining independently generated biological components while retaining their capacity for self-organization. Early examples included so-called “complex organoids” in which human iPSC-derived mesodermal progenitors were combined with neural or tumor organoids, resulting in integrated vascular, connective-tissue, and tissue-resident macrophage or microglial compartments. In neuroscience, Birey and colleagues demonstrated that dorsal and ventral forebrain spheroids could be fused to reconstruct interneuron migration and circuit formation [20]. Subsequent nomenclature frameworks defined assembloids as self-organizing three-dimensional systems generated by combining an organoid with another organoid or with specialized cell types, followed by integration of the assembled components [30,31,32,33,34,35,36,37,38,39].

Importantly, assembly strategy and functional validation represent distinct axes and should not be conflated. Assembly strategy describes how the model is constructed and may include organoid–organoid fusion (multi-region), integration of an organoid with additional specialized cell lineages (multi-lineage), or incorporation of organizer-like populations [38,39]. Functional validation instead addresses what emerges after assembly: evidence may include formation of a biologically relevant tissue–tissue interface, lineage-specific interactions, cell migration, signaling across compartments, or emergent tissue-level functions that are not reproduced by the individual components alone. Structural integration and self-organization are considered separately as evidence that the assembled components have formed an integrated 3D system. Thus, simple juxtaposition or co-maintenance of separately generated components, even in three dimensions, is more appropriately described as a co-culture or multicellular 3D model when subsequent integration and self-organization have not been demonstrated. Likewise, microfluidic control or perfusion defines an organ-on-chip engineering format rather than, by itself, an assembloid; a chip may contain an organoid, co-culture, or independently validated assembloid.

This distinction is particularly important in reproductive biology, where the term “assembloid” has been applied to models with substantially different degrees of integration. A foundational example is the endometrial system developed by Rawlings and colleagues, in which gland-like epithelial organoids were co-embedded with primary endometrial stromal cells in a physiologically tuned collagen hydrogel [25]. Following assembly, the epithelial and stromal compartments self-organized into a gland-in-stroma architecture resembling key features of native endometrium and, upon hormonal stimulation, generated differentiated and senescent epithelial and stromal states characteristic of implantation-window endometrium, together with decidual secretory responses [25]. These observations provide evidence of both structural integration and emergent functional behavior, supporting its designation as an endometrial assembloid rather than simply an epithelial–stromal co-culture.

Throughout this review, we therefore classify reproductive models along two independent axes: assembly strategy and functional validation (Table 1). We use “assembloid” when the reported system combines an organoid with another organoid or specialized lineage and demonstrates subsequent three-dimensional integration and self-organization. We use “co-culture” or “multicellular 3D model” when components are combined but evidence of such integration is limited or has not been demonstrated, and “organ-on-chip” when the defining feature is engineered microfluidic control. Functional validation is considered separately from this structural classification: outputs including decidualization, embryo attachment or invasion, cilia-driven transport, and endocrine responses are described as experimentally demonstrated properties that establish what the assembled system can model, rather than determining its platform identity. This framework is applied consistently to the reproductive systems discussed in Section 3.

### 2.3. A Working Nomenclature and Adjacent Systems

Because the reproductive literature applies “assembloid” to systems with differing assembly strategies and degrees of integration, we use the criterion-based terminology established above. Assembly strategy and functional validation are considered independently. Following established assembloid nomenclature, assembly strategies may include multi-region systems generated by fusion of regionally distinct organoids and multi-lineage systems generated by combining organoids with additional specialized cell populations; other engineered configurations may impose spatial, morphogen, or layered organization depending on the biological question [39]. In reproductive biology, epithelial–stromal configurations predominate, while incorporation of immune, endothelial, smooth-muscle, or other specialized populations remains model- and protocol-specific. Five related but distinct platforms are compared according to their assembly strategy, integration criteria, representative components, functional validation, and limitations in Table 1.

For the purposes of this review, reproductive 3D models are assigned a platform designation primarily according to their mode of assembly and evidence of post-assembly integration and self-organization; cellular composition and experimentally demonstrated function are reported separately. Organoids are self-organizing three-dimensional structures derived from tissue-specific stem/progenitor cells or pluripotent stem cells that reproduce selected architectural and functional features of the modeled tissue or developmental unit. We reserve the term assembloid for systems in which an organoid is combined with another organoid or with one or more specialized cell populations and the components subsequently demonstrate three-dimensional integration and self-organization, rather than merely coexist in culture. Depending on their construction, these may include multi-region assembloids generated by fusion of independently patterned organoid modules and multi-lineage assembloids generated by incorporation of additional stromal, immune, endothelial, smooth-muscle, or other specialized lineages. Spatially organized or layered configurations are described according to their architecture but are designated as assembloids only when the same post-assembly integration criteria are met. Importantly, the designation “assembloid” does not imply the presence of all native tissue lineages: many reproductive assembloids currently consist primarily of epithelial and stromal compartments, whereas immune or endothelial incorporation remains protocol-specific.

**Figure 1:**
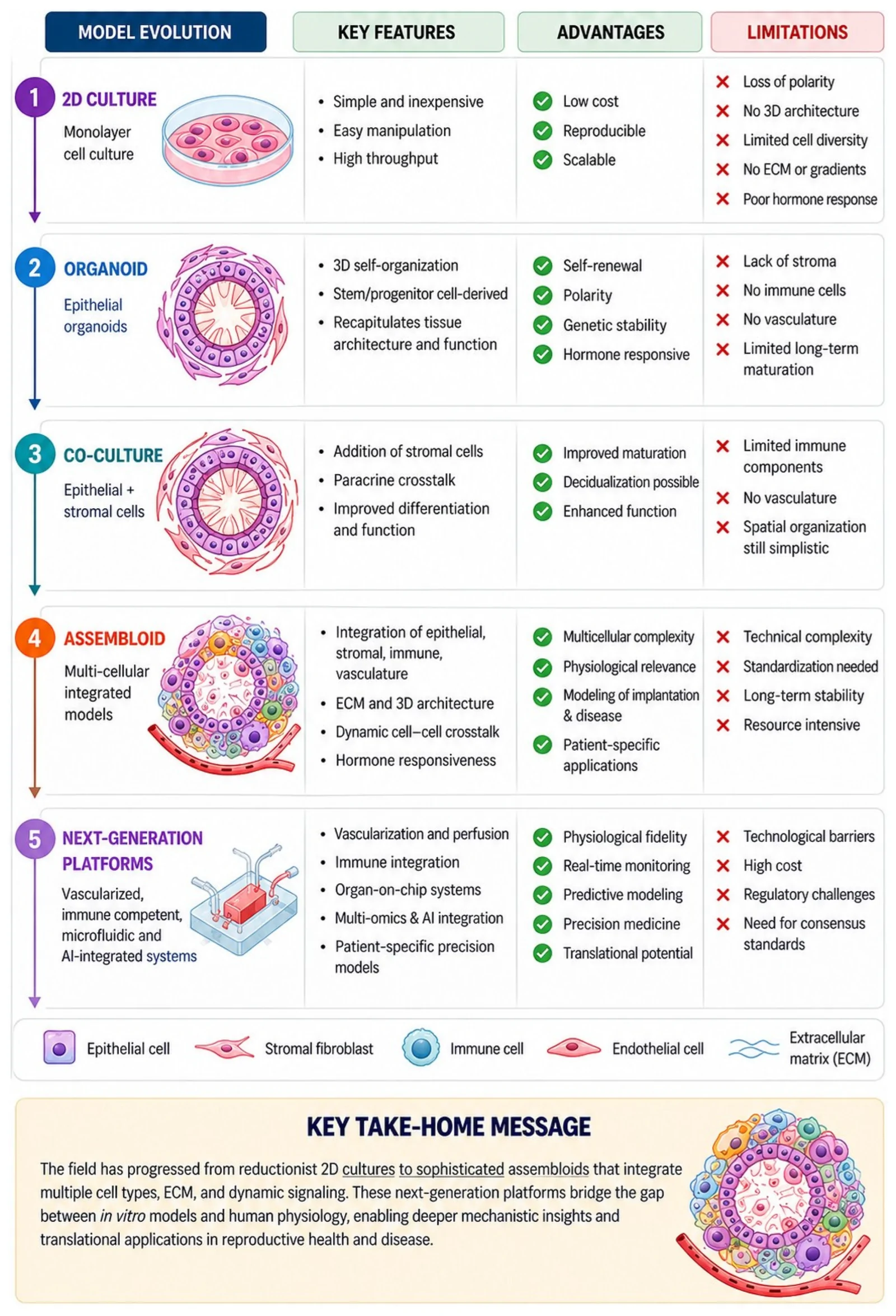
Major experimental model classes in female reproductive biology and their complementary dimensions of complexity. Schematic overview of conventional two-dimensional (2D) cultures, organoids, co-cultures and multicellular 3D models, assembloids, and engineered organ-on-chip platforms. These model classes differ in their assembly strategy, cellular composition, degree of post-assembly integration, and engineering control rather than representing a strictly linear developmental hierarchy. Increasing biological complexity may involve incorporation of stromal, immune, vascular, and other specialized compartments, whereas engineering complexity may include extracellular-matrix control, perfusion, microfluidics, live imaging, and multi-omics integration. Individual platforms may combine these features according to the biological question being addressed. Green check marks indicate advantages or desirable attributes of each model, whereas red crosses indicate limitations or challenges.

By contrast, we use “co-culture” or “multicellular 3D model” for systems in which separately generated cell types or modules are maintained together but post-assembly integration and self-organization are limited or have not been demonstrated. Functional interaction alone does not determine platform identity; conversely, an assembloid need not reproduce every function of the native tissue. Organ-on-chip systems are classified separately because their defining feature is engineered microfluidic control, although they may incorporate organoids, co-cultures, multicellular 3D models, or independently validated assembloids. Blastoids are stem-cell-derived models of the preimplantation embryo and constitute a distinct embryo-model category rather than a subtype of reproductive organoid or maternal assembloid. When combined experimentally with an endometrial model, the maternal and embryonic components are therefore classified independently according to their respective construction and integration criteria. The relationships among these model classes and their increasing biological and engineering complexity are summarized in Figure 1.

### 2.4. Design Principles: Compartments, Matrix, and Cell Source

Three design choices strongly influence the architecture and functional performance of reproductive multicellular 3D models: the compartments incorporated, the extracellular matrix, and the cellular source. These variables affect model behavior but are conceptually distinct from the classification criteria used above to define an assembloid. The first is which compartments to integrate. In endometrial models, epithelial–stromal integration is central to reconstructing decidualization and receptivity-associated interactions. In a gland-morphogenesis model, direct epithelial–stromal co-embedding supported organoid viability and gland-tube formation, whereas conditioned medium alone did not reproduce these effects, demonstrating that physical stromal contact was required in that experimental system [40,41,42,43,44,45,46,47,48,49,50]. Adding immune cells, including uNK cells, macrophages, and regulatory T cells, may extend these models to investigate immune tolerance, inflammatory signaling, and senescent-cell clearance during receptivity [47,51,52], while endothelial incorporation offers a route to model vascular interactions and, when coupled to appropriate perfusion strategies, may help address diffusion limitations and enable investigation of trophoblast–vascular remodeling.

The second is the extracellular matrix. Basement-membrane extracts favor cystic epithelial growth, whereas collagen-rich or tuned composite matrices can support stromal migration, tissue compaction, and mechanically distinct microenvironments [25,48]; fully synthetic, defined matrices allow matrix composition and biochemical cues to be controlled while interrogating epithelial–stromal interactions [30]. Spatial organization, including gland–stroma architecture and, in implantation-oriented models, accessibility of a luminal-like epithelial surface, depends on both matrix properties and assembly strategy. Matrix selection should therefore be considered an experimental design variable rather than a defining feature of any particular model class.

The third is cell source. Primary tissue-derived models preserve donor-specific genetic backgrounds and can retain disease-associated phenotypes, making them particularly valuable for patient-specific endometrial modeling, although their use is constrained by tissue availability, donor-to-donor variability, and potential differences introduced during culture. Pluripotent stem cell (PSC)-derived components, including PSC-derived endometrial stromal fibroblasts capable of cyclic hormone-responsive decidualization [42] and Müllerian duct-like progenitors with epithelial and stromal differentiation potential [43], offer complementary routes for generating reproductive cell types when primary tissue is limited and for modeling genetically defined conditions. However, developmental maturity, differentiation fidelity, and epigenetic state remain important considerations for PSC-derived systems. Hybrid approaches combining primary and PSC-derived components may provide additional experimental flexibility, although their degree of integration and functional validation should be assessed using the same criteria applied to other multicellular models. These design strategies are considered again in the organ-specific sections below.

## 3. Applications Across the Female Reproductive System

Throughout this section, models are designated as organoids, co-cultures, multicellular 3D models, assembloids, or organ-on-chip systems according to the framework defined in Section 2. Where relevant to the biological question, we benchmark each model according to the tissue compartments represented, evidence of direct intercompartmental interaction, reconstruction or accessibility of a luminal epithelial surface, incorporation of immune and vascular components, and experimentally demonstrated functional outputs. These features are used to assess model capability and biological completeness rather than to define assembloid identity. The term “assembloid” is reserved for systems in which post-assembly integration and self-organization have been demonstrated; where evidence for such integration is limited or incomplete, we use the more conservative term “co-culture” or “multicellular 3D model,” or retain the terminology used by the original authors with appropriate qualification. Each model is considered according to both its assembly strategy and its experimentally demonstrated structural and functional properties.

**Figure 2:**
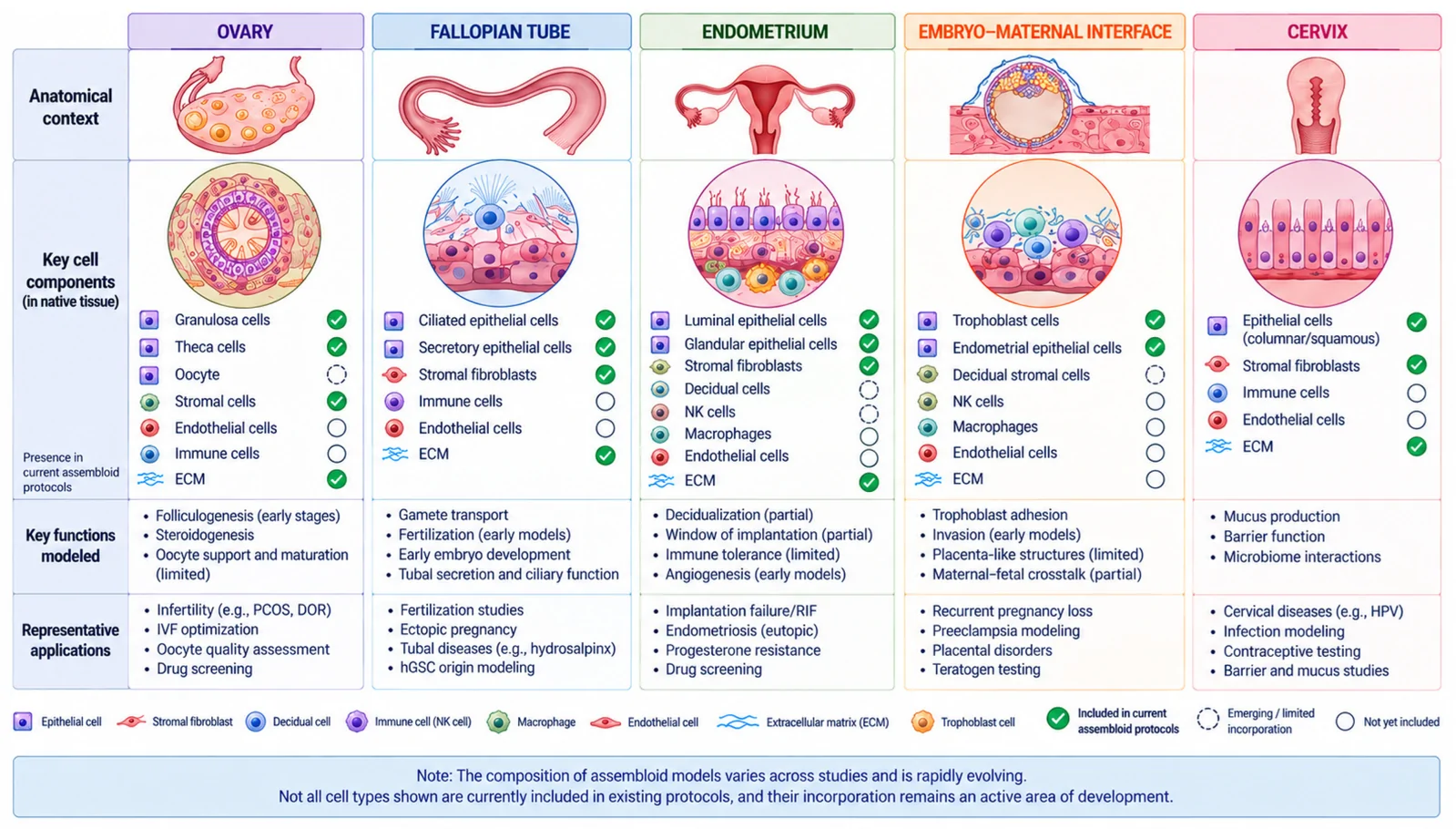
Cellular composition of female reproductive 3D models: native tissue context and current modeling status. Schematic overview of organ-specific reproductive models across the ovary, fallopian tube, endometrium, embryo–maternal interface, and cervix. Cell types shown represent major components of the corresponding native tissues, whereas symbols indicate their current level of incorporation into reported assembloid or related multicellular 3D protocols (included, emerging/limited, or not yet incorporated). Importantly, model composition varies substantially among studies, and no individual platform necessarily contains all cell types depicted. Key functions currently modeled and representative experimental applications are summarized for each compartment.

The following sections survey, organ by organ, what current reproductive 3D models have demonstrated, integrating disease modeling and the relevant enabling technologies into each compartment and reserving cross-cutting engineering and limitations for Section 3.5. The endometrium is treated at greatest length because it represents the most mature reproductive 3D modeling system; the ovary, fallopian tube, and embryo–maternal interface follow, encompassing platforms at different stages of development. The principal cellular components, modeled functions, and emerging applications of these reproductive 3D models across anatomical compartments are summarized in Figure 2.

### 3.1. The Endometrium: The Most Mature Assembloid System

#### 3.1.1. Epithelial–Stromal Assembloids and Gland Morphogenesis

Endometrial epithelial–stromal models combine glandular epithelial organoids with primary endometrial stromal fibroblasts (ESFs) in physiologically informed matrices, restoring aspects of the paracrine and biophysical crosstalk absent from organoid monocultures [25,34]. When these components undergo post-assembly spatial integration and self-organization, as demonstrated in the foundational Rawlings system [25], they meet the criteria for designation as endometrial assembloids. Building on this framework, several complementary designs with different degrees of structural and functional integration have been reported.

An “instant assembloid” combines organoids and ESFs in collagen I under a condensed three-day hormonal protocol (E2, a progestin, 8-bromo-cAMP), inducing canonical stromal decidual markers (PRL, SCARA5, DIO2) alongside epithelial secretory genes (PAEP, SPP1) and supporting short-term human blastocyst attachment; the collagen I matrix promotes stromal migration and tissue compaction and yields a rapid, scalable protocol [48]. A dedicated gland-morphogenesis assembloid combining endometrial organoids with human endometrial stromal cells in a collagen–Matrigel scaffold self-organizes into lumenised tubular glands within seven days, and demonstrates that direct stromal contact, not conditioned medium, is required for epithelial viability and tube formation, mechanistically tied to estradiol-driven suppression of WNT7B [50]. An organotypic epithelial–stromal model using conditionally reprogrammed epithelium overlaid on hormonally stimulated stroma, reproduce a transient autoinflammatory decidual response and induce the epithelial receptivity marker integrin αvβ3 in the overlying epithelium [53,54].

A parallel line of work has restored the luminal epithelium that glandular-only assembloids omit. Air–liquid-interface models embed ESFs in a collagen-rich matrix and overlay organoid-derived glandular epithelium, which self-organizes at the air-exposed apical surface into a polarized, MUC1-expressing luminal-like epithelium bearing receptivity markers on hormone stimulation [31]. An equivalent mouse system built on an optimized Matrigel–collagen composite with a defined 1:4 glandular-to-stromal ratio generates a MUC1/ZO-1-positive luminal layer, clusters transcriptomically with in vivo endometrium, and recapitulates the pre-receptive-to-receptive transition, including upregulation of Msx1, Pax2 and Gata2, providing a genetically tractable receptivity platform [23]. Fully synthetic-matrix co-cultures, in turn, allow epithelial–stromal crosstalk to be interrogated under defined biochemical conditions [30]. Collectively, these complementary models demonstrate how stromal integration, direct epithelial–stromal contact, and reconstruction of an accessible luminal-like surface can each address specific limitations of epithelial organoids, although these features are not uniformly represented across individual platforms.

#### 3.1.2. Decidualization and Decidual Senescence

Decidualization represents one of the most extensively characterized functional outputs of endometrial assembloids. Single-cell profiling of decidualized assembloids resolves the stroma into differentiated decidual and senescent decidual subpopulations, the latter marked by p21/p16 signatures and SASP factors (IL-6, IL-8, IGFBP1, CXCL family), alongside differentiated and senescent epithelial states [25]. Importantly, decidual senescence appears to have a functional role in supporting embryo–endometrial interactions rather than representing merely a byproduct of differentiation. Human blastocysts co-cultured with decidualized assembloids expanded and gained motility, whereas the senolytic dasatinib selectively eliminated senescent decidual cells and restricted embryo growth [25]. These findings provide functional evidence linking stromal senescence to an implantation-supportive decidual environment, a relationship that cannot readily be interrogated in epithelial-only organoids or conventional 2D decidualization assays.

At the compartment level, epithelial acute senescence enhances the secretion of implantation-associated factors, whereas stromal senescence contributes to the balance between anti-inflammatory and pro-inflammatory decidual states [25]. In the cycling endometrium, this balance is maintained in part through uNK-mediated clearance of senescent decidual cells, an interaction that immune-integrated endometrial models are beginning to capture [47]. Cyclic modeling further extends this concept: subjecting instant assembloids to repeated decidualization–withdrawal cycles shows that acute, cyclic senescence preserves progenitor pools and construct architecture, whereas prolonged decidualization increases stress-associated markers, reduces stemness, and compromises assembloid integrity [53]. Collectively, these findings support senescence as a physiological and dynamically regulated component of endometrial receptivity rather than an exclusively pathological process.

#### 3.1.3. The Window of Implantation and Immune Integration

Reconstruction of the receptive mid-secretory endometrium and incorporation of immune populations represent important frontiers in endometrial assembloid development. A receptive endometrial assembloid integrating epithelial, stromal, and immune compartments, with glands surrounded by stromal and immune populations, has been used to interrogate the window of implantation [55]. In this model, the receptive state was associated with altered energy metabolism, including larger and functionally active mitochondria, increased ATP production, and elevated IL-8, linking metabolic remodeling to decidual competence [55].

Immune integration is particularly relevant because uNK cells contribute to the clearance of senescent decidual cells in the cycling endometrium [47], while implantation-associated immune tolerance involves coordinated interactions among uNK cells, macrophages, and regulatory T-cell populations that are not captured by classical decidual markers alone [52]. Immune-integrated assembloids capable of reproducing these interactions may therefore provide a platform for investigating the contribution of immune–stromal–epithelial crosstalk to endometrial receptivity and its disruption. Importantly, the emergence of such models demonstrates that immune-cell incorporation is feasible but remains protocol-specific rather than an intrinsic feature of endometrial assembloids (Section 3.5).

#### 3.1.4. Progesterone Resistance, Endometriosis, and Adenomyosis

Because patient-derived multicellular models can retain disease-associated features across epithelial and stromal compartments, they enable aspects of stromal pathology and epithelial–stromal interactions that are not captured by epithelial organoids alone. A patient-derived endometriosis assembloid, composed of glandular epithelium and stromal cells, forms spherical constructs approximately 1.15 mm in diameter and expresses the epithelial marker cytokeratin and the stromal marker CD10. When applied to a reconstructed peritoneal surface, these assembloids adhered to the recipient tissue and recapitulated features of early superficial peritoneal lesion establishment. CD10-positive stromal cells infiltrated the peritoneal wall to a depth of approximately 341 µm, demonstrating the model’s ability to reproduce adhesion and stromal invasion during early endometriosis establishment in a fully patient-derived system [26].

For adenomyosis, multi-layer assembloids combining epithelial organoids with endometrial stromal and smooth-muscle cells from normal, eutopic, and ectopic tissue recapitulate glandular invasion into the smooth-muscle compartment at high resolution [9], whereas epithelial–stromal assembloids from adenomyosis patients model impaired endometrial receptivity associated with progesterone resistance [37]. A developmental counterpart to this disease work showed that multicellular uterine models combining neonatal uterine epithelium with different mesenchymal sources direct divergent epithelial cell fates, drawing a mechanistic link between developmental epithelial plasticity and oncogenic reprogramming [22]. Collectively, these systems provide experimentally accessible functional readouts of disease-associated phenotypes, including progesterone resistance, impaired receptivity, and endometriotic adhesion and invasion, complementing molecular and expression-based observations.

#### 3.1.5. Recurrent Implantation Failure and Recurrent Pregnancy Loss

Endometrial multicellular models have provided new mechanistic insights into endometrial dysfunction associated with recurrent implantation failure (RIF) and recurrent pregnancy loss (RPL). Modeling the sequelae of iterative decidual stress shows that repeated decidual inflammatory cycles deplete endometrial stem cells and drive progesterone resistance, providing an in vitro correlate of the progressive decidual dysfunction associated with recurrent miscarriage [5]. The accompanying clinical analysis suggests that RPL risk may reflect not only embryonic factors but also a measurable disruption of decidual progression, specifically the time-sensitive depletion of progesterone-resistant DIO2+ stromal cells that form the implantation niche and the failure to expand progesterone-dependent PLA2G2A+ predecidual cells, changes that were detectable before conception [5]. Cyclic-senescence assembloids similarly model progressive impairment of endometrial regenerative capacity, a mechanism proposed to contribute to endometrial dysfunction in some patients with RIF following repeated ovarian stimulation [53].

Reviews of endometrial-organoid applications to infertility identify receptivity assessment, personalized embryo-transfer timing, and drug-sensitivity testing as potential translational applications; the incorporation of the stromal compartment in assembloid models may further extend these approaches by enabling assessment of epithelial–stromal interactions [46]. Collectively, these studies support an endometrial contribution to RIF and RPL and provide experimentally tractable models for investigating disease-associated decidual and regenerative dysfunction.

More recently, this framework has been extended from molecular and cellular phenotyping to patient-specific functional implantation modeling. In a 3D in-chip system, blastoids showed significantly reduced implantation capacity in endometrioids derived from patients with RIF compared with those derived from fertile controls, providing a direct functional readout of patient-specific endometrial implantation competence [56,57,58,59,60,61,62,63,64].

#### 3.1.6. Patient-Derived Precision Medicine and Drug Screening

The patient specificity that makes reproductive organoids and multicellular 3D models valuable for disease modeling also creates opportunities for individualized drug testing. Long-term patient-derived endometrial organoids already show clinically informative, individualised responses to progesterone, antiprogestins such as mifepristone, and MEK inhibitors in endometrial cancer, supporting their potential as patient-specific experimental avatars [56]. Incorporating stromal and other tissue compartments may extend this capability by revealing decidualization-dependent and compartment-specific drug responses that cannot be assessed in epithelial monoculture. In integrated epithelial–stromal models, compounds may differentially affect stromal decidualization, epithelial hormone responsiveness, or reciprocal signaling between these compartments [35,49]. Such context-dependent responses may be particularly relevant to disorders involving altered epithelial–stromal signaling, including endometriosis, adenomyosis, and abnormal uterine bleeding.

PSC-derived stromal and Müllerian components further enable modeling when primary patient tissue is limited or unavailable, including in the context of Mayer–Rokitansky–Küster–Hauser syndrome, uterine malformations, and genetic ovarian insufficiency [42,43]. More recently, the use of patient-specific reproductive 3D models for therapeutic testing has begun to move from conceptual potential toward experimental proof-of-concept. Patient-derived RIF endometrioids coupled to blastoids have been used as functional implantation assays and as platforms for targeted screening of FDA-approved compounds, identifying candidate interventions that increased implantation efficiency in RIF-derived endometrioids [64]. Such systems provide proof-of-concept that patient-derived implantation models can couple functional assessment of endometrial competence with individualized therapeutic screening, although prospective clinical validation will be required before these approaches can inform patient management.

### 3.2. The Ovary and Follicle

#### 3.2.1. Follicle Organoids and PSC-Derived Folliculogenesis

Ovarian modeling is substantially less mature than endometrial modeling, and much of the current literature is more appropriately positioned as being “toward” assembloid development rather than representing established assembloid systems. Two major complementary approaches have been pursued. Primary follicular cells can be grown in three dimensions to preserve follicular architecture and support oocyte growth and meiotic maturation beyond what is typically achievable in conventional 2D culture [24]. In parallel, PSC-based approaches can generate primordial germ cell-like cells (PGCLCs) through signaling pathways involving BMP4/WNT3A and induction of germ-cell specification factors including PRDM1, PRDM14, and TFAP2C, while directed differentiation can generate granulosa-like cells that respond to FSH and produce E2 and P4 [57,58,60]. In mice, PSC-derived germ cells have been progressed through oogenesis to generate functional oocytes capable of producing live offspring [57]; achieving an equivalent system in humans remains substantially more challenging and will require not only an appropriate somatic niche capable of supporting germ-cell development and meiotic progression, but also faithful epigenetic reprogramming and demonstration of developmental competence.

#### 3.2.2. Granulosa–Theca–Oocyte Crosstalk and the Artificial Ovary

The follicle is intrinsically a multi-lineage unit, and reconstituting reciprocal signaling among granulosa cells, theca cells, and the oocyte represents a major challenge in reconstructing follicular function in vitro. Self-assembly approaches, including the organization of theca-like cells around granulosa spheroids or cumulus–oocyte complexes, yield honeycomb-like microtissues that support oocyte maturation [59,60]. More complex reconstituted ovarian models can also self-organize from PSC-derived somatic and germ-cell components, demonstrating that multicellular ovarian architecture can emerge from defined cellular populations without the obligatory inclusion of ovarian surface epithelium [59,60]. “Artificial ovary” constructs that encapsulate ovarian or mesenchymal support cells have been developed to provide endocrine support following transplantation and have partially restored ovarian endocrine function in rodent models [62,63]. However, adequate vascularization remains a major barrier to the long-term survival and function of transplanted constructs, prompting strategies including prevascularization and delivery of pro-angiogenic factors [62].

#### 3.2.3. Hormone Secretion, Oocyte Maturation, and IVF

A practical goal is the generation of mature, developmentally competent oocytes in vitro, with potential future applications for patients with premature ovarian insufficiency and survivors of gonadotoxic treatment. Current clinical in vitro maturation of cumulus–oocyte complexes aspirated from antral follicles yields lower blastocyst rates than conventional IVF, in part because nuclear maturation may not be accompanied by complete cytoplasmic maturation [58]. Emerging multicellular ovarian models seek to reconstruct key components of the follicular niche and its endocrine function. Measurable granulosa-cell steroidogenesis, including E2 and P4 production, has been demonstrated in transcription-factor-driven ovaroid systems [60]. When such ovarian modules are functionally coupled to an endometrial compartment, their hormone production can elicit phase-appropriate endometrial responses, providing a model of the ovarian–endometrial endocrine axis across interconnected tissue compartments [60]. However, structural reconstruction and steroidogenic function alone do not establish oocyte developmental competence. Faithful meiotic progression and epigenetic reprogramming remain major unresolved challenges, particularly for translation of PSC-derived ovarian models to human gametogenesis.

#### 3.2.4. Toward In Vitro Gametogenesis

In vitro gametogenesis (IVG), the derivation of developmentally competent gametes from PSCs in vitro, represents a potentially transformative but still highly experimental frontier in reproductive biology [57,60]. Human progress currently reaches PSC-derived germ-cell-like cells that require appropriate somatic support for further differentiation and meiotic progression; transcription-factor-driven granulosa-like co-cultures have supported early meiotic-prophase progression of human PGCLCs and, in selected experimental systems, the generation of zona-bearing oocyte-like cells [60]. Progress toward complete human IVG will therefore require reconstruction of multiple features of the gonadal niche, including appropriate somatic-cell interactions, temporally regulated developmental signals, and extracellular-matrix cues. Multicellular ovarian models incorporating these elements may provide an experimental bridge between PSC differentiation and the generation of functionally competent human gametes, although this goal has not yet been achieved.

The potential clinical translation of IVG, however, raises substantial ethical, legal, and societal concerns that extend beyond its technical feasibility. Most immediately, the genetic and epigenetic fidelity of IVG-derived gametes and the absence of long-term safety data raise concerns regarding potentially heritable risks to future offspring. Moreover, the capacity to generate large numbers of gametes, and consequently embryos, could substantially expand embryo selection and genetic screening, raising questions regarding enhancement, eugenic applications, and the ethical status and disposition of surplus embryos. Derivation of gametes from somatic cells also introduces distinctive issues of informed consent and unauthorized parenthood, while potential applications to same-sex reproduction, solo reproduction, and reproduction at advanced ages may challenge existing legal and societal concepts of parenthood and reproductive autonomy. Finally, high costs and restricted availability could exacerbate existing inequities in access to assisted reproduction. Accordingly, clinical translation of human IVG will require not only rigorous demonstration of genetic, epigenetic, and developmental safety but also transparent societal dialogue and dedicated regulatory frameworks addressing consent, reproductive justice, embryo use, and the welfare of future offspring [65,66,67].

### 3.3. The Fallopian Tube

The fallopian tube provides a specialized microenvironment for fertilization and early embryo transport and secretes factors that support gamete and early embryo development. Its mucosa is lined by a simple columnar epithelium composed predominantly of ciliated and secretory cells, supported by stromal and smooth-muscle compartments. Long-term fallopian tube organoids retain ciliary beating, secretory function, and hormone responsiveness and therefore provide an epithelial component from which more complex multicellular tubal models can be constructed [32]. Single-cell studies have identified a WNT7A–FZD5 axis that maintains fallopian tube stem cells in patient-derived organoids, providing mechanistic insight into the maintenance of these cultures [27].

A more complex multicompartment fallopian tube model integrates ciliated, secretory, stromal, and smooth-muscle populations using a tissue-map-guided assembly strategy informed by global label-free proteomics and whole-organ single-cell transcriptomic mapping [8]. Importantly, the resulting construct demonstrated spatial organization of these cellular compartments together with cilia-driven transport of oocyte-mimicking particles, providing both structural and functional evidence of post-assembly integration and supporting its classification as a fallopian tube assembloid under the criteria used in this review [8]. This tissue-map-guided approach illustrates how native cellular composition and spatial organization can inform assembloid design, although further refinement of multicompartment architecture and functional maturation remains necessary.

A more complex multicompartment fallopian tube model integrates ciliated, secretory, stromal, and smooth-muscle populations using a tissue-map-guided assembly strategy informed by global label-free proteomics and whole-organ single-cell transcriptomic mapping [8]. Importantly, the resulting construct demonstrated spatial organization of these cellular compartments together with cilia-driven transport of oocyte-mimicking particles, providing both structural and functional evidence of post-assembly integration and supporting its classification as a fallopian tube assembloid under the criteria used in this review [8]. The demonstrated transport function, however, was based on surrogate particles rather than direct oocyte or embryo transport, and functional contractility of the smooth-muscle compartment was not established. Thus, the model is notable for its multicompartment tubal architecture and cilia-associated transport readout, while coordinated muscular transport and gamete/embryo transit remain to be demonstrated.

Two disease applications are particularly relevant to future tubal modeling. The fallopian tube, particularly the fimbrial epithelium, is a major site of origin for HGSOC, with many cases developing through precursor lesions including serous tubal intraepithelial carcinoma (STIC) [33]. Existing epithelial organoid studies can model selected tumor-associated genetic alterations, but they do not by themselves reconstruct the normal stromal and smooth-muscle microenvironment of the tube. Accordingly, extending such disease models into multicellular tubal systems remains a prospective application rather than an established capability of current fallopian tube assembloids. Multicellular models incorporating genetically altered or patient-derived secretory epithelial cells together with relevant stromal compartments may in future provide experimental systems for investigating TP53-driven transformation, STIC progression, and early epithelial–stromal interactions during invasion [8,33].

### 3.4. The Embryo–Maternal Interface

#### 3.4.1. Blastoids and Trophoblast Organoids

The peri-implantation interface has historically been difficult to model because of the limited availability of human embryos and the ethical and regulatory considerations surrounding their use; blastoids and trophoblast organoids now provide complementary experimental systems for investigating embryo- and trophoblast-side biology. Naïve-PSC-derived blastoids reproduce key features of blastocyst organization and contain cell populations resembling the three principal blastocyst lineages: trophectoderm (GATA3), epiblast (OCT4), and hypoblast (SOX17), with lineage-associated activity of Hippo, Wnt, FGF, and BMP signaling pathways [40,61]. Their scalable generation enables quantitative implantation and screening experiments that would be difficult to perform using human embryos; however, transcriptomic differences from natural blastocysts persist, including in genes associated with zygotic genome activation and early epigenetic reprogramming [40,61]. Accessible microwell and small-molecule protocols have further increased the scalability and experimental accessibility of blastoid generation, enabling larger and more standardized implantation assays [40]. In parallel, self-renewing trophoblast organoids model key features of early placental trophoblast differentiation and can generate differentiated trophoblast populations, including syncytiotrophoblast and extravillous trophoblast (EVT), providing renewable material for mechanistic studies and co-culture experiments [18,19].

#### 3.4.2. Integrated Embryo–Endometrial Implantation Models

A major functional benchmark for endometrial models is their ability to reproduce sequential embryo–endometrial interactions, including apposition, adhesion, and invasion. This has been modeled using apical-out endometrial–mesenchymal organoids, which present the receptive luminal surface outward, within a collagen-rich stroma containing a self-organized endothelial network. Combining these with human blastoids produced integrated embryo–maternal constructs that sequentially reproduced apposition, adhesion, invasion, syncytiotrophoblast formation, and trophoblast–stromal fusion. Experiments using natural human blastocysts further demonstrated hCG secretion, trophoblast-marker induction, and syncytial gene expression [10].

More recently, two complementary human implantation platforms have substantially extended the experimental scope of embryo–endometrial modeling. Li et al. developed a 3D in-chip implantation model in which human blastoids or blastocysts were co-cultured with bioengineered human endometrial tissue (“endometrioids”), supporting implantation and early post-implantation developmental events. Importantly, patient-derived endometrioids from individuals with RIF showed reduced implantation capacity, while screening of FDA-approved compounds identified candidate interventions that increased implantation efficiency in RIF-derived endometrioids [64]. In parallel, Molè et al. reconstructed the luminal, glandular, and stromal compartments of receptive human endometrium and demonstrated implantation of both human embryos and blastoids, followed by early post-implantation development and trophoblast differentiation. Single-cell profiling of the day-14 embryo–endometrial interface resolved conceptus–maternal signaling networks, while experimental perturbation of extravillous trophoblast–stromal signaling reduced trophoblast outgrowth, providing functional evidence for reciprocal embryo–endometrial communication [65].

Together, these models extend embryo–endometrial modeling beyond morphological reconstruction by enabling experimental analysis of intercompartmental signaling, patient-specific implantation phenotypes, and, in selected systems, therapeutic response. Importantly, they employ distinct assembly strategies and should not be considered interchangeable: some demonstrate post-assembly tissue integration consistent with an assembloid framework, whereas others are more appropriately classified as engineered co-culture or in-chip implantation platforms. Reviews have similarly emphasized that pairing blastoids with physiologically timed endometrial models offers an experimental strategy for investigating contributions from both embryonic and maternal compartments to implantation failure [34,35].

#### 3.4.3. Integrated Platforms, Microfluidics, and Post-Implantation Modeling

Microfluidic embryo–endometrium platforms add flow, compartmentalisation, and live imaging to static co-culture, allowing controlled exchange across the embryo–maternal interface and real-time visualization of implantation-related events [44]. Live-imaging approaches established in mouse systems provide a methodological framework for analogous human studies, although their application remains constrained by the limited availability and regulated use of human embryos [44]. Combining scalable blastoid generation with such platforms may enable more standardized and higher-throughput implantation assays [40].

Post-implantation events, including EVT invasion, vascular remodeling, and syncytialisation, can also be interrogated using engineered microphysiological systems. One such platform quantifies trophoblast invasion depth and MMP secretion and identifies TGFβ1 and IL-6 as modulators of invasive behavior, while permitting pharmacological perturbation with anti-adhesion or MMP-inhibitory compounds [41]. In a distinct assembly strategy, the feto-maternal assembloid provides a three-dimensional stromal context in which trophoblast–stromal fusion and syncytial formation can be observed [10]. These complementary systems therefore model different aspects of post-implantation biology rather than representing a single platform class.

A remaining challenge is the incorporation of functional, remodelable vascular compartments that can interact directly with invading trophoblast. Such vascularized multicellular models could enable more physiologically representative investigation of EVT–vascular interactions and spiral-artery remodeling and may ultimately provide experimental platforms for studying their disruption in disorders such as preeclampsia and fetal growth restriction.

#### 3.4.4. Ethical and Regulatory Considerations

Because these systems model increasingly complex aspects of early human development and embryo–maternal interactions, they fall within an evolving ethical and regulatory landscape. Blastoids and integrated embryo–maternal models raise questions about the extent to which stem-cell-derived embryo models approximate features of natural human embryos, how such models should be classified and governed, and what limits should apply to their developmental progression in vitro [34,36]. These considerations become particularly relevant as embryo models are combined with increasingly physiologically representative endometrial tissues that support implantation-like and post-implantation events. Transparent reporting of each model’s cellular composition, developmental potential, degree of correspondence to natural embryos, and experimental endpoint, together with adherence to applicable regulatory requirements and evolving international professional guidelines, is essential for responsible progress and meaningful ethical oversight.

### 3.5. Enabling Technologies and Current Limitations

#### 3.5.1. Microfluidics, Organ-on-Chip, and Multi-Organ Integration

Organ-on-chip systems provide engineering capabilities that complement organoid and assembloid models, including controlled continuous or pulsatile flow, compartmentalized paracrine signaling, live imaging, and functional coupling between tissue modules [44,45]. A multi-organ microfluidic platform linked murine ovary with human fallopian tube, endometrial, cervical, and liver modules under actuated flow and modeled endocrine interactions over a 28-day reproductive cycle, with phase-dependent E2 and P4 production by the ovarian module [21]. This study demonstrated the feasibility of functionally coupling multiple reproductive and metabolic tissue compartments within a single microfluidic system. Endometrium-on-chip devices can similarly combine epithelial, stromal, and vascular compartments under controlled flow, enabling dynamic interrogation of tissue–tissue and tissue–vascular interactions [44,45]. Importantly, the organ-on-chip format describes the engineering environment rather than the biological classification of the tissue model it contains; accordingly, a microfluidic platform may incorporate organoids, co-cultures, or independently validated assembloids. Integrating a structurally and functionally validated multicompartment model with controlled perfusion therefore offers a complementary strategy for combining biological self-organization with dynamic environmental control [8].

#### 3.5.2. Vascularization

Vascularization is a major unresolved engineering challenge for increasingly complex reproductive 3D models. Without adequate vascular perfusion, diffusion-limited oxygen and nutrient delivery constrains construct size and may restrict the spatial gradients and multicellular interactions required to model decidualization and trophoblast invasion [49]. Encouragingly, endothelial networks can self-organise within multicellular reproductive models under appropriate matrix and cell-density conditions, as demonstrated by the spontaneous endothelial network in the feto-maternal assembloid [10]. Current strategies include co-embedding endothelial cells to promote self-organizing vascular networks, generating perfusable channels through bioprinting or sacrificial templating, and using microfluidic perfusion to provide controlled flow; these approaches may also be combined. Coupling self-organizing endothelial networks with microfluidic perfusion offers one strategy for introducing both biological vascular organization and controlled flow, whereas bioprinting and templating approaches may ultimately permit greater geometric control over vessel architecture [49]. Further development of perfusable, remodelable vascular compartments could enable more physiologically representative modeling of EVT–vascular interactions, including spiral-artery remodeling, and their disruption in pregnancy disorders.

#### 3.5.3. Hydrogels, Matrix, and Bioprinting

Matrix choice strongly influences construct architecture, mechanical properties, cellular differentiation, hormone responsiveness, and long-term stability. Increasingly, matrix design is being tailored to the requirements of individual tissue compartments. Collagen-rich matrices can provide stromal support, whereas defined synthetic matrices permit controlled interrogation of epithelial–stromal interactions [30]. Decellularized tissue-specific ECM represents a complementary strategy by retaining aspects of the native biochemical and structural microenvironment while removing cellular components [48]. Bioprinting provides an engineering approach for controlling construct geometry, spatial cell placement, and the design of perfusable architectures beyond what can be specified through biological self-organization alone [49]. For example, alginate-based bioinks offer tunable crosslinking and favorable printing properties but provide limited intrinsic cell-adhesive cues; the incorporation of adhesion-promoting components, such as RGD-containing motifs or fibronectin-derived elements, can improve cell–matrix interactions [49]. Combining biologically instructive matrices with spatially controlled fabrication may therefore help reconcile the self-organizing properties of reproductive tissues with the structural reproducibility required for increasingly complex multicompartment models.

#### 3.5.4. Single-Cell and Spatial Benchmarking, and Live Imaging

Single-cell and spatial transcriptomics have become important benchmarking tools for complex reproductive 3D models, enabling comparison of model-derived cell states with in vivo reference tissues while also identifying emergent cellular states and intercellular signaling programs. Single-cell profiling resolved differentiated and senescent epithelial and stromal states in endometrial assembloids and identified senescence-associated communication networks [25], while whole-organ single-cell mapping combined with proteomics provided a native-tissue reference for the design and validation of the multicompartment fallopian tube assembloid [8]. Rather than functioning solely as descriptive technologies, single-cell and spatial approaches can therefore provide a reference framework for assessing cellular identity, composition, and spatial organization and for guiding iterative model refinement. Where appropriate native-tissue atlases are available, they can inform both the design of multicellular constructs and the evaluation of their correspondence to in vivo tissues. Live imaging provides a complementary temporal dimension, allowing dynamic processes such as decidualization, gland morphogenesis, and embryo–endometrial interactions to be followed over time. Combined with quantitative image analysis, these approaches may also facilitate high-content phenotyping and future automated screening applications.

#### 3.5.5. Reproductive Toxicology

A distinct translational application of reproductive 3D models is their potential use in reproductive toxicology. The endometrium and ovary are sensitive targets for endocrine-disrupting chemicals (EDCs), heavy metals, and environmental pollutants, while species-specific differences in reproductive physiology can limit the direct translation of findings from conventional animal models to humans [51,52]. Human multi-organ platforms that integrate endocrine-responsive reproductive tissues may complement single-organ assays by capturing inter-tissue endocrine signaling and systemic responses to reproductive toxicants. Such platforms could be particularly informative for EDCs whose effects extend across multiple hormonally responsive compartments. Patient-derived multicellular models of disorders such as endometriosis could further provide a framework for investigating whether environmental exposures interact with disease-associated cellular phenotypes or modify individual susceptibility [51,52]. Future incorporation of immune populations may additionally enable the assessment of exposure-related changes in immune–stromal–epithelial interactions that are not captured by conventional molecular or decidualization readouts.

#### 3.5.6. Critical Limitations

Despite rapid progress, several limitations continue to constrain the physiological fidelity, reproducibility, and translational potential of reproductive 3D models. Vascularization remains incompletely represented in most systems, although self-organizing endothelial networks have been demonstrated in selected models and multiple engineering strategies are being explored to establish functional perfusion [10,49]. Immune-cell incorporation has been reported in selected endometrial models [55] but remains protocol-specific and incompletely characterized. The immune populations represented, their abundance and persistence within the constructs, and the extent of functional validation vary among models. Accordingly, the common limitation that reproductive 3D models incompletely represent the native immune compartment requires qualification rather than being considered resolved. Notably, foundational epithelial–stromal endometrial assembloids, including the Rawlings model [25], do not themselves incorporate immune cells. Long-term stability and phenotypic maturation also remain important challenges, particularly in PSC-derived and ovarian models, where faithful meiotic progression, epigenetic reprogramming, and developmental competence remain incompletely resolved. Finally, standardization, reproducibility, and scalability remain limited: matrices, cell ratios, hormonal protocols, and functional readouts differ between laboratories, while dependence on primary cells introduces donor-to-donor variability, complicating cross-study comparison, protocol harmonization, and eventual clinical qualification. Addressing these limitations will require coordinated advances in biological model design, engineering, benchmarking, and standardization, together with application-specific validation before reproductive 3D models can be translated into robust experimental or clinical platforms.

## 4. Concluding Remarks and Perspectives

Within roughly a decade, female reproductive modeling has moved from predominantly single-lineage epithelial organoids toward increasingly complex multicellular and multicompartment systems with varying degrees of post-assembly integration. These platforms have enabled modeling of decidualization and its senescence dynamics at single-cell resolution [25], sequential embryo–endometrial interactions including attachment, invasion, and trophoblast differentiation [10], PSC-based reconstruction of ovarian somatic and germ-cell compartments, with functional gametogenesis achieved in mouse systems [57], tissue-map-guided construction and validation of a multicompartment fallopian tube assembloid using whole-organ proteomic and transcriptomic references [8], and microfluidic coupling of multiple reproductive tissue modules to model endocrine crosstalk [21]. The diversity of these systems—endometrial, ovarian, tubal, and embryo–maternal; static and microfluidic; primary and PSC-derived; patient-specific and experimentally standardized that reflects a field advancing on several fronts while increasingly drawing on shared enabling approaches, including native-tissue-guided design, single-cell and spatial benchmarking, engineered matrices and microfluidics, and dynamic imaging.

The translational potential of these models spans both near- and longer-term applications, although most remain experimental and will require prospective validation before clinical implementation. Patient-derived reproductive 3D models can provide platforms for individualized disease modeling and therapeutic screening, while recent embryo–endometrial systems have demonstrated the feasibility of measuring patient-specific implantation phenotypes and testing candidate interventions in RIF-derived endometrial models [64]. Such approaches may ultimately contribute to functional assessment of endometrial receptivity and to the development of individualized therapeutic strategies, but their ability to improve clinical decision-making, embryo-transfer outcomes, or treatment selection remains to be established. Related platforms may also facilitate investigation of endometrial preparation, progesterone responsiveness, and candidate secreted biomarkers [48]. On a longer horizon, vascularized artificial-ovary strategies may provide new approaches to endocrine and fertility restoration after gonadotoxic treatment [62], while human IVG, if developmental competence and genetic and epigenetic safety can be established and the associated ethical, legal, and societal challenges are addressed within appropriate regulatory frameworks, may eventually expand reproductive options for individuals who cannot otherwise generate functional gametes.

**Figure 3:**
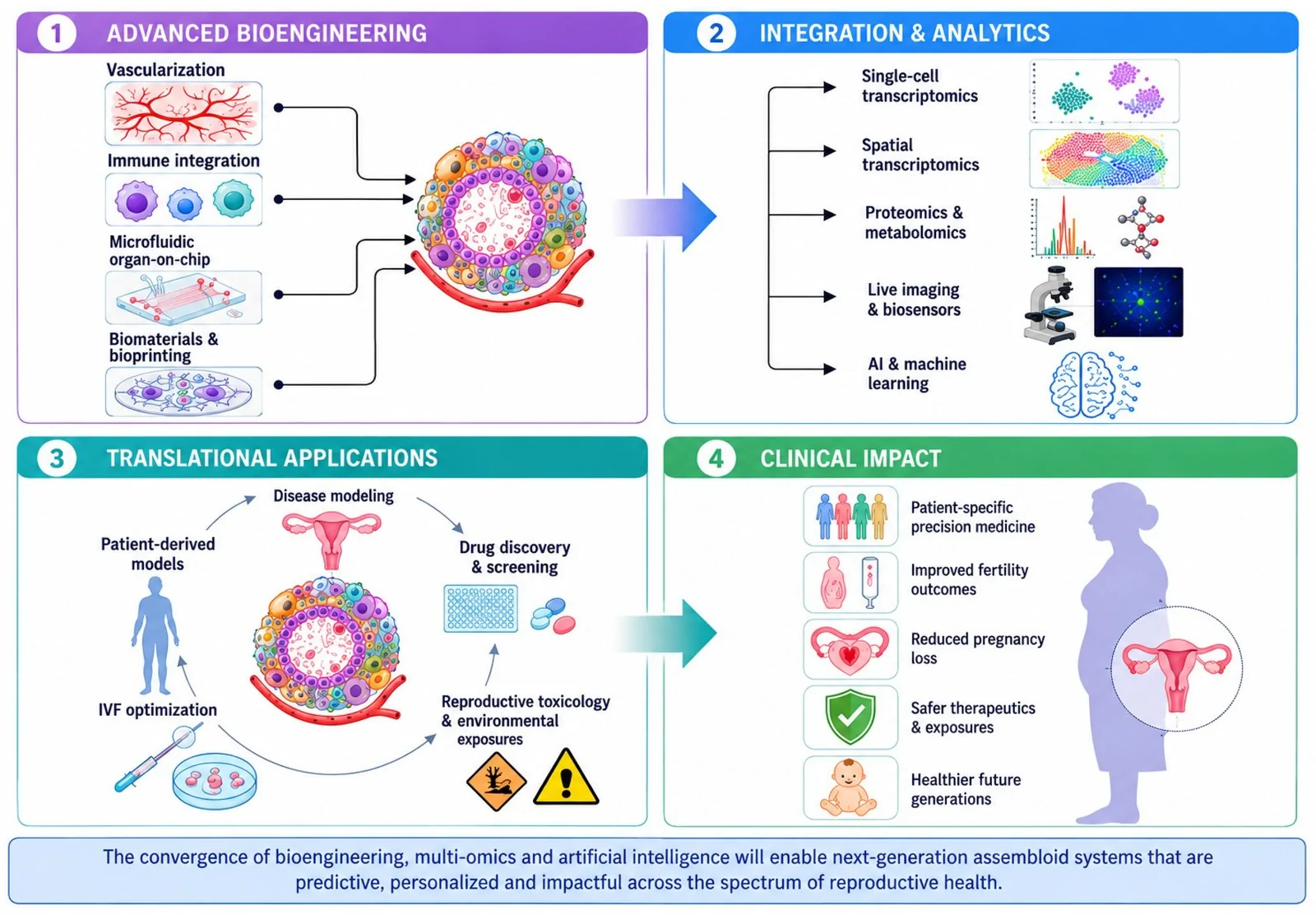
Future roadmap toward personalized and predictive reproductive medicine. Conceptual roadmap for the development and potential translation of next-generation reproductive 3D models and integrated platforms. Advances in vascularization, immune integration, microfluidic organ-on-chip technologies, biomaterials, and bioprinting may increasingly converge with single-cell and spatial transcriptomics, proteomics and metabolomics, live imaging, biosensors, and computational analytics. These complementary technologies may support increasingly physiologically representative and quantitatively benchmarked models for patient-derived disease modeling, drug discovery and screening, assisted-reproduction research, and reproductive toxicology. With appropriate biological and technical validation, standardization, and ethical and regulatory oversight, such platforms may ultimately contribute to more personalized approaches to reproductive medicine and to improved assessment of therapeutic and environmental effects on reproductive health.

Realising this potential will depend on addressing the limitations outlined above, including incomplete vascularization and immune representation, limited maturation and long-term stability, protocol heterogeneity, and challenges in standardization and reproducibility, together with appropriate ethical and regulatory oversight of embryo models and integrated embryo–maternal systems. Several complementary directions are likely to shape further development. Integrating biologically validated multicompartment models with microfluidic perfusion may enable more dynamic and physiologically representative vascular, immune, and endocrine interactions without conflating the organ-on-chip format with assembloid identity. In parallel, single-cell and spatial profiling, quantitative live imaging, and computational analysis can provide increasingly rigorous benchmarks of cellular identity, tissue organization, and functional response and may support reproducible high-content phenotyping and therapeutic screening [49]. The convergence of these bioengineering, multi-omics, computational, and translational advances may progressively increase the physiological fidelity and experimental utility of reproductive 3D models and support their future development toward more personalized approaches in reproductive medicine, as illustrated in Figure 3.

The studies reviewed here establish a strong foundation for increasingly sophisticated human reproductive 3D models, while also highlighting the biological, engineering, and translational challenges that remain. By integrating tissue-specific cellular complexity with dynamic environmental control and rigorous molecular and functional benchmarking, these platforms offer opportunities to investigate implantation failure, endometriosis, progesterone resistance, ovarian dysfunction, and other reproductive disorders in experimentally tractable human systems. Their ultimate clinical value, however, will depend on demonstrating reproducibility, physiological fidelity, predictive validity, and safety in the context of each intended application. Continued convergence of reproductive biology, bioengineering, single-cell and spatial technologies, and computational analysis may progressively bridge mechanistic discovery and clinically relevant modeling, while preserving clear distinctions among organoids, assembloids, co-cultures, and engineered organ-on-chip platforms.

## Figures and Tables

**Table 1:** Criterion-based comparison of major three-dimensional reproductive modeling platforms.

Platform	Assembly Strategy	Integration Criterion	Representative Components	Functional Validation	Key Limitations
Organoids	Self-organization from tissue-specific stem/progenitor cells or PSCs	Intrinsic self-organization within a single tissue or developmental unit	Tissue-specific epithelial or other organ-specific lineages	Model-dependent tissue architecture, polarity, differentiation, secretion, or hormone responsiveness	Limited representation of heterotypic tissue compartments
Assembloids	Fusion of independently patterned organoids or integration of an organoid with specialized cell lineage(s)	Post-assembly 3D integration/self-organization and formation of a biologically relevant interface	Model-dependent; organoid(s) + stromal, immune, endothelial, smooth-muscle, or other specialized lineage(s)	Model-dependent emergent tissue-level behavior demonstrated after assembly	Variable integration and maturation; incomplete lineage representation; protocol heterogeneity
Co-culture/multicellular 3D model	Co-maintenance of two or more independently generated cell types or modules	Spatial integration/self-organization absent, limited, or not demonstrated	Epithelial +/− stromal; trophoblast +/− maternal cells; other paired/multiple populations	Paracrine signaling, attachment/invasion, matrix remodeling, or other assay-specific responses	Limited or unproven tissue-level organization and emergent behavior
Organ-on-chip	Engineered microfluidic compartmentalization with controlled flow and/or mechanical cues	Determined by the biological model incorporated; the chip format itself does not establish assembloid integration	Epithelium +/− stroma +/− endothelium +/− immune or other tissue modules	Flow-dependent responses, dynamic signaling, live interface analysis, or multi-tissue coupling	Engineering complexity, low throughput, and standardization challenges
Blastoids	PSC-directed self-organization into a blastocyst-like structure	Self-organized embryo-like lineage architecture	EPI-like, TE-like, and PE-like lineages	Blastocyst-stage development and embryo-side biology; implantation interactions when combined with maternal models	Developmental differences from natural blastocysts; ethical and regulatory constraints

## Data Availability

No new data were created or analyzed in this study. Data sharing is not applicable to this article.
